# Differential regulation of synaptic AP-2/clathrin vesicle uncoating in synaptic plasticity

**DOI:** 10.1038/s41598-017-16055-4

**Published:** 2017-11-17

**Authors:** Ermes Candiello, Ratnakar Mishra, Bernhard Schmidt, Olaf Jahn, Peter Schu

**Affiliations:** 1 Department of Cellular Biochemistry, University Medical Center Goettingen, Georg-August-University Göttingen, Humboldtallee 23, 37073 Göttingen, Germany; 2 The Max-Planck-Institute for Experimental Medicine, Proteomics, Hermann-Rein-Str. 3, 37073 Göttingen, Germany

## Abstract

AP-1/σ1B-deficiency causes X-linked intellectual disability. AP-1/σ1B −/− mice have impaired synaptic vesicle recycling, fewer synaptic vesicles and enhanced endosome maturation mediated by AP-1/σ1A. Despite defects in synaptic vesicle recycling synapses contain two times more endocytic AP-2 clathrin-coated vesicles. We demonstrate increased formation of two classes of AP-2/clathrin coated vesicles. One which uncoats readily and a second with a stabilised clathrin coat. Coat stabilisation is mediated by three molecular mechanisms: reduced recruitment of Hsc70 and synaptojanin1 and enhanced μ2/AP-2 phosphorylation and activation. Stabilised AP-2 vesicles are enriched in the structural active zone proteins Git1 and stonin2 and synapses contain more Git1. Endocytosis of the synaptic vesicle exocytosis regulating Munc13 isoforms are differentially effected. Regulation of synaptic protein endocytosis by the differential stability of AP-2/clathrin coats is a novel molecular mechanism of synaptic plasticity.

## Introduction

AP-1 and AP-2 clathrin adaptor-protein complexes have essential functions in synaptic vesicle (SV) recycling. AP-2 clathrin-mediated-endocytosis (CME) facilitates SV endocytosis, AP-1 complexes mediate TGN/endosome protein sorting via clathrin-coated-vesicles (CCV). In brain two γ1AP-1 complexes exist, which share β1 and μ1A, but differ in their σ1 adaptins. The ubiquitous AP-1 contains σ1A, the tissue-specific AP-1 contains σ1B. Deficiency of the X-chromosome encoded σ1B causes severe mental retardation^1^. AP-1/σ1B knock-out synapses show alterations in AP-1 dependent SV protein sorting and transport, but also in CME^1,2^. They have slower and incomplete SV recycling, while early endosomes become enlarged. AP-1/σ1A accumulates on them and activates the Rab5/Vps34 PI 3-kinase (PI3KCIII) pathway stimulating their maturation into multivesicular endosomes. AP-1/σ1B inhibits this AP-1/σ1A function^3^. A fraction of SV proteins accumulates in early endosomes, whereas others are degraded^2^. Electron microscopy had demonstrated accumulation of synaptic CCV and their biochemical analysis revealed that they are AP-2 CCV instead of malformed AP-1 CCV^1,2^. Synapses contain 100% more AP-2 CCV and 80% more AP-2 than wt synapses. σ1B −/− cortices contain only 30% more AP-2 than wt cortices. This increase was unexpected given that SV recycling, the major synaptic vesicular transport route, is impaired.

σ1B-deficiency induced changes in AP-1 and AP-2 dependent protein transport are brain and synapse specific, as demonstrated by the analysis of σ1B −/− adipose tissue. σ1B −/− mice have a lipodystrophy due to enhanced inhibition of adipogenesis. Inhibition is caused by sortilin missorting and its associated overexpression. σ1A and σ1B have overlapping cargo protein binding specificities, but only σ1B binds sortilin. Sortilin levels are not increased in σ1B −/− brains^4,5^. γ1AP-1 levels are reduced in σ1B −/− synapses, but not in adipocytes, and AP-2 levels are only increased by 10% in σ1B −/− adipocytes^6^. Thus the changes in AP-1 and AP-2 dependent protein transport in σ1B −/− synapses are synapse specific and are thus novel molecular mechanisms of synaptic plasticity.

AP-2 CCV are mainly formed at the plasma membrane. However, during fast endocytosis of SV, plasma membrane is internalised first by actin mediated endocytosis to form plasma membrane vacuoles. Then, AP-2 forms CCV from these vacuoles^7–9^. Thus reduced SV recycling should not lead to an increase in AP-2 CCV. This increase could be caused by the stimulation of CME of synaptic proteins other than SV proteins and/or by a slowed down uncoating of AP-2 CCV, extending their half life. If CME is upregulated, the question arises, which synaptic proteins are endocytosed and why they are endocytosed via this slow, protein selective route, instead of the less selective, but faster bulk membrane endocytic pathways^7,9,10^. If the protein coat of these CCV is more stable and their uncoating delayed, all possible downstream events will be slowed down. Slower protein transport to acceptor organelles, e.g. early endosomes, would slow down recycling of SV proteins or of other synaptic plasma membrane proteins (receptors, ion channels, transporters). It would delay signal transduction pathways of internalised receptors. Alterations in AP-2 CME is an adaptation to the impaired AP-1 dependent SV recycling and may be able to suppress its consequences on trans-synaptic signalling.

In order to gain insight into this AP-2/clathrin dependent mechanism of synaptic plasticity, we characterised the synaptic AP-2 CCV biochemically and compared the protein composition of AP-2 CCV isolated from σ1B −/− and wt mice. We demonstrate upregulation of CME and the existence of a novel AP-2 CME pathway, which operates in parallel to the classic AP-2 CME pathway. The novel AP-2 CCV are stabilised by three different molecular mechanisms, demonstrating complex regulatory mechanisms. They are enriched in structural synaptic active zone (AZ) proteins and thus have a function in AZ plasticity. This is a novel molecular mechanism of synaptic plasticity. They also reveal that AP-2/clathrin mediated endocytosis fulfils functions beyond the simple removal of proteins from the cell surface.

## Results

### AP-2 CCV stability

We isolated synaptic CCV following the established sucrose density gradient centrifugation protocol. There was no difference in the fractionation of AP-2 isolated from wt and σ1B −/− synapses on the gradient. In our first semi-quantitative western-blot analysis we had determined an 80% increase in synaptic AP-2 and a 120% increase in synaptic AP-2 CCV^2^. The clathrin basket forms the outer layer of CCV. The clathrin-heavy-chain (CHC) forms triskelia, which connect via their proximal and distal legs building hexagonal and pentagonal lattices. Basket disassembly requires Hsc70, which is recruited to its site of action by its co-chaperones auxilin-1 and −2. However, Hsc70 has additional basket binding sites^11–21^. In our previous analysis we found no increase in Hsc70 in σ1B −/− synapses, but a 100% increase in synaptic CCV associated Hsc70 compared to wt CCV^2^. Thus the majority of AP-2 CCV in σ1B −/− synapses undergo rapid uncoating similar to wt CCV and the increase in AP-2 CCV could be solely due to enhanced CME, despite the reduction in SV recycling. Could the AP-2 CCV half life be extended as well, due to delayed uncoating of the CCV? Activated Hsc70 binds CHC in a dynamic way and mediates basket disassembly by permanent ‘collision pressure’ against the basket from its inside. If Hsc70 is not guided to the basket uncoating domain, it will bind comparably stably and promiscuously to other CCV protein domains^11^. We reasoned that if a stabilised AP-2 CCV pool exists in σ1B −/− synapses, those CCV should have a different, stable mode of Hsc70 binding, which might allow their separation from the total CCV pool.

**Figure 1:**
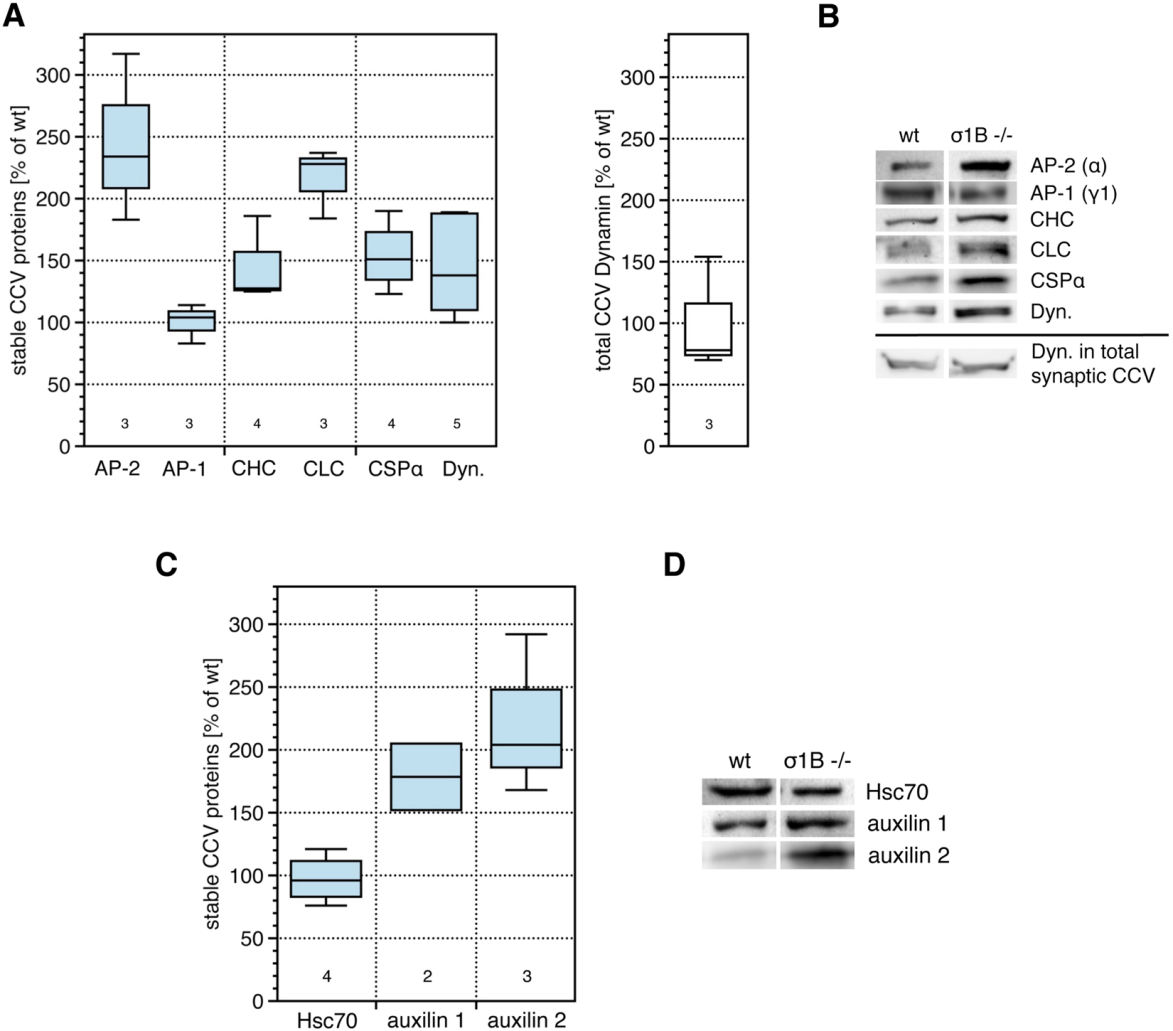
Classification of the immunoisolated, stable CCV from σ1B −/− and wt synapses by comparing their coat protein composition. Data are presented as box-plot diagrams and show the quantification of independently prepared biological samples (wt = 100%). Numbers given below the box plots indicate the independently performed experiments. (**A**) Analysis of coat proteins in stabilised CCV are shown as blue boxes. The comparison of the dynamin content in the total synaptic CCV pool is shown as a white box. (**B**) Representative semi-quantitative western-blot images used in the quantification shown in A (please also refer to Mat. & Meth.). (**C**) Comparison of the association of the clathrin basket disassembling Hsc70 and its two co-chaperones with the immunoisolated, stable CCV from σ1B −/− and wt synapses. (**D**) Representative images of the semi-quantitative western-blots analysis used for the quantification are shown in C (please also refer to Mat. & Meth.).

For the analysis of CCV stability one has to be aware, that their protein coat is not a rigid protein network. CCV are unstable in physiological buffers. CCV isolation requires slightly acidic pH in order to stabilise their coat. Raising the pH to slightly alkaline pH afterwards is sufficient to induce coat disassembly^22–24^. We first isolated synaptic CCV at pH 6.4 on a sucrose gradient following the established protocol^2,25^. Next we performed an anti-Hsc70 CCV immunoisolation from the pooled CCV gradient fractions. Antibodies isolated CCV from σ1B −/− synapses and importantly also from wt synapses. Immunoisolated CCV from σ1B −/− synapses contain two times more AP-2 than the corresponding pool from wt mice, similar to the AP-2 ko/wt ratio found in the total synaptic CCV pool. We also compared the AP-1 content of these CCV from wt and σ1B −/− synapses and found it to be identical (Fig. 1A,B). Thus AP-1 CCV formed in σ1B −/− synapses are not stabilised compared to the corresponding wt CCV. The molecular mechanisms responsible for CCV half life extension have to be specific for AP-2 CCV and this particular endocytic pathway.

Immunoisolated CCV from σ1B −/− synapses have slightly more CHC, but they contain two times more clathrin-light-chains (CLC) (Fig. 1A,B). CLC bind to the proximal legs and take part in the regulation of basket stability and life time^14^. Tissues vary in their CHC/CLC ratios. Only in brain is the ratio 1, while other tissues contain less CLC than CHC. The increase in CLC indicates that these CCV from σ1B −/− synapses are indeed stabilised compared to those from wt synapses. The large GTPase dynamin is essential for the last step in CCV formation. It assembles into rings, constricts the vesicle neck and detaches CCV from the plasma membrane^26,27^. CCV immunoisolated from σ1B −/− contain more dynamin than the respective wt CCV. The total CCV pool of σ1B −/− synapses contains less dynamin than wt CCV, which would be in line with a fast CCV uncoating reaction (Fig. 1A,B). This increase is in line with slow dynamin ring disassembly and thus slow CCV uncoating.

CSPα is a presynapse specific chaperone known to be incorporated into CCV^28–30^. Previously we have shown that synaptic CCV from σ1B −/− synapses contain more of it than wt CCV^2^. Also the immunoisolated CCV contain more CSPα than the corresponding wt CCV, but the increase is comparable with the increase in the total σ1B −/− synapse CCV pool^2^ (Fig. 1A,B). Thus CSPα does not play a role in CCV stabilisation.

Next we analysed whether immunoisolated CCV from σ1B −/− synapses have less of Hsc70 and auxilin-1/−2 than those CCV from wt synapses. In our previous analysis we detected an increase in Hsc70 in the total synaptic CCV pool which matches the increase in AP-2^2^. However there is no difference in the amount of Hsc70 associated with CCV immunoisolated from wt and σ1B −/− synapses, despite the increase in AP-2 CCV. This reduced AP-2:Hsc70 ratio and reduction in Hsc70 is in line with a stabilised pool of AP-2 CCV (Fig. 1C,D). Surprisingly, more auxilin-1/2 co-chaperones are associated with CCV from σ1B −/− synapses (Fig. 1C,D). Regulatory mechanisms apparently prevent Hsc70 activation by auxilin-1/−2 and Hsc70 binds to CCV sites, at which it is not undergoing ADP-ATP cycles and does not disassemble the clathrin basket^11^.

Firstly, these data demonstrate the existence of a more stable AP-2 CCV pool within a synapse. Secondly, they demonstrate that this AP-2 CCV pool is increased and more stabilised in σ1B −/− than in wt synapses. Next we tested whether also AP-2 membrane binding is stabilised.

### AP-2 hyperactivation

**Figure 2:**
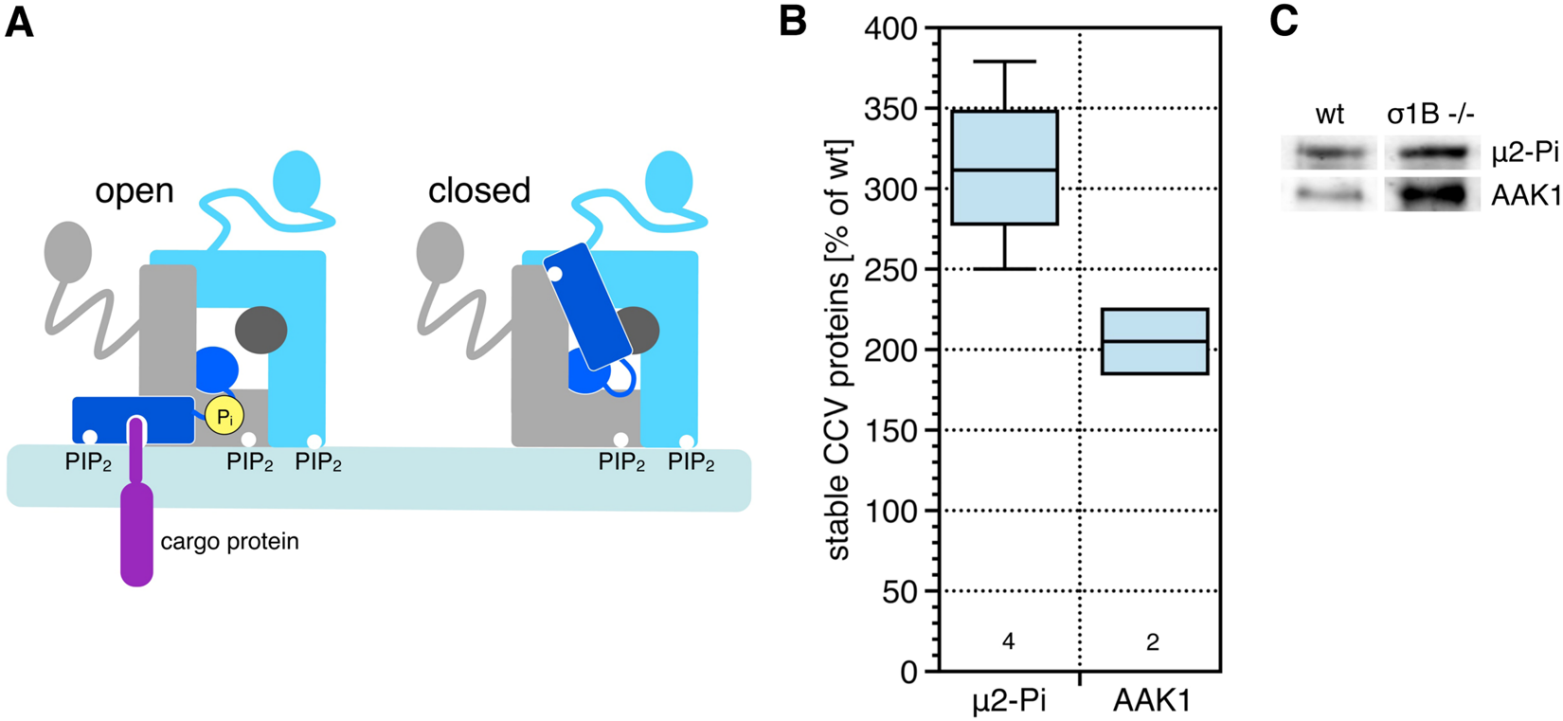
(**A**) Cartoon illustrating the increase of the AP-2 membrane binding affinity by μ2 phosphorylation (μ2 dark blue, α light blue, β2 grey, σ2 dark grey). (**B**) Comparison of the amount of μ2 adaptin phosphorylation and AAK1 kinase association with the immunoisolated, stable CCV from σ1B −/− and wt synapses. Quantification of semi-quantitative western-blot signals of independently prepared biological samples (wt = 100%). Numbers given below the box plots indicate the independently performed experiments. (**C**) Representative images of the western-blots of the analysis shown in A (see also Math. & Meth.).

AP-2 recruits clathrin to the membrane and the CCV life cycle is controlled via the regulation of AP-2 membrane binding. High affinity AP-2 membrane binding requires its binding to cargo proteins and to PI-4,5-P_2_ (PIP_2_). These interactions are regulated and require a conformational change of AP-2. This reorientation releases a steric blockade of the cargo protein binding domains in μ2 and σ2 adaptins and brings the PIP_2_ binding motif of μ2 in proximity to the membrane. This transition is supported by μ2-Thr^156^ phosphorylation by the AAK1 kinase (Fig. 2A). AAK1 is not the only kinase phosphorylating μ2, but these kinases have not yet been identified^31–35^. We determined the amount of activated AP-2 complexes using a μ2-Thr^156^-Pi specific antiserum^33,36^. This revealed an increase of μ2-Pi by 35% in the total synaptic CCV pool of σ1B −/− synapses compared to wt CCV in our previous study^2^. This is less than expected given the increase in AP-2, which again suggests a higher turnover rate of the majority of AP-2 CCV in σ1B −/− synapses compared to wt synapses. Also less AAK1 kinase is associated with these CCV compared to wt CCV^2^. In immunoisolated CCV from σ1B −/− the μ2-Pi level is increased by 215% compared to the wt CCV. AAK1 is increased by 100% in these CCV compared to the corresponding wt CCV (Fig. 2B,C). This is a second molecular mechanism for the differential stabilisation of these AP-2 CCV.

### Less synaptojanin-1 recruitment

**Figure 3:**
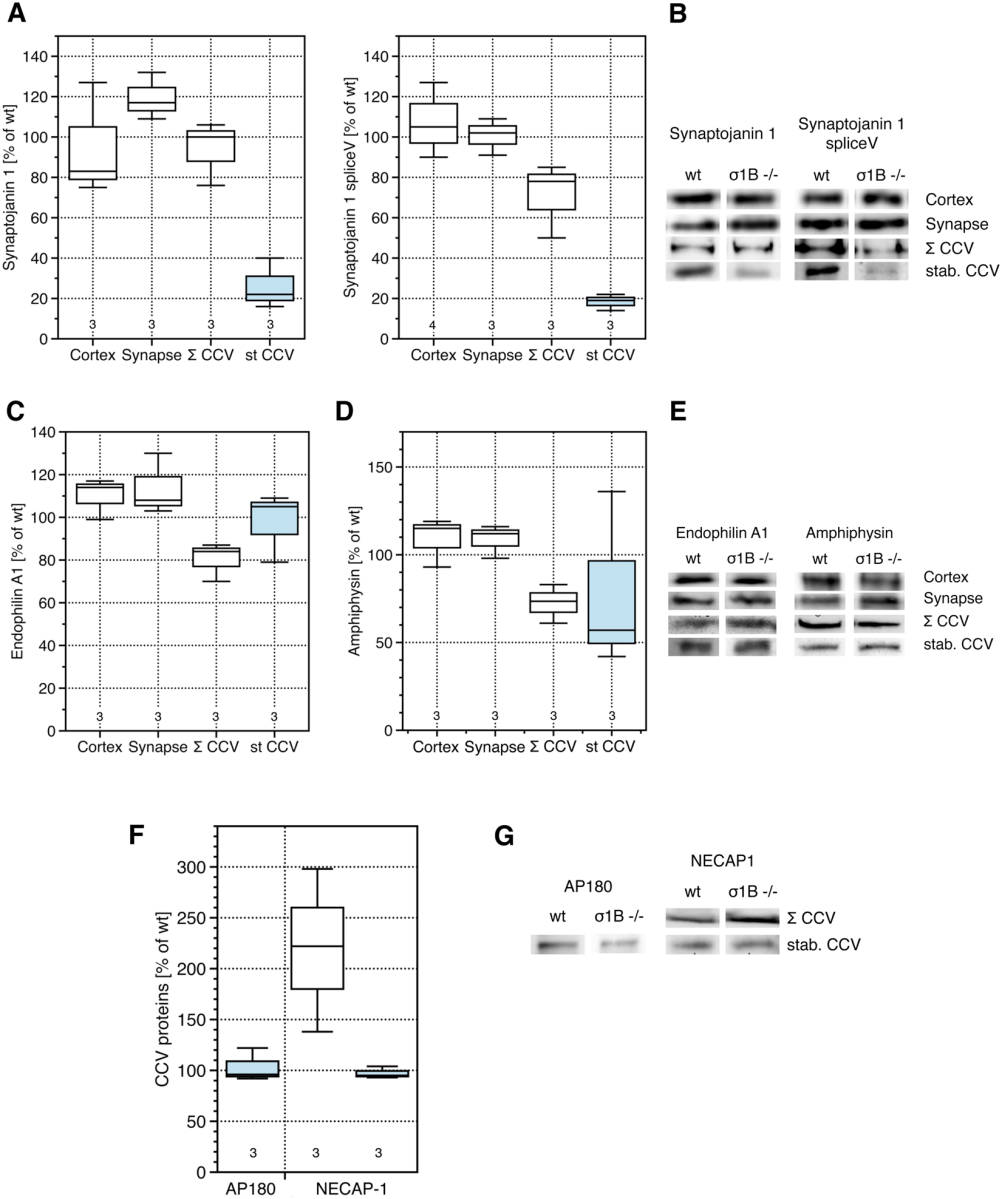
(**A**) Comparison of the expression and the distribution of synaptojanin-1 and its splice variant in σ1B −/− and wt cortices and of their association with the total pool of synaptic CCV (Σ CCV) and with the immunoisolated, stable CCV (st CCV, blue boxes) from σ1B −/− and wt synapses. (**B**) Representative western-blots of the analysis shown in A. (**C**) Comparison of the expression and the distribution of endophilin A1 in σ1B −/− and wt cortices and of its association with the total pool of synaptic CCV and with the stable CCV from σ1B −/− and wt synapses. (**D**) Association of amphiphysin with the total pool of synaptic CCV and with the stable CCV from σ1B −/− and wt synapses. (**E**) Representative western-blots of the analysis shown in (**C** and **D**). Box-plot diagrams show the quantification of semi-quantitative western-blots of independently prepared biological samples (wt = 100%). Numbers given below the box plots indicate the independently performed experiments. (**F**) Comparison of the association of the co-adaptor proteins AP180 and NECAP-1 with the stable CCV (st CCV, blue box) and synaptic CCV (Σ CCV, white box) from σ1B −/− and wt synapses. Quantification of semi-quantitative western-blots of independently prepared biological samples (wt = 100%). Numbers given below the box plots indicate the independently performed experiments. (**G**) Representative western-blots of the analysis shown in A.

AP-2 membrane dissociation requires PIP_2_ phosphatase activities of ubiquitous synaptojanin-1, and of the brain-specific splice variant with a C-terminal truncation^37^. σ1B −/− cortices contain less synaptojanin-1, but synapses contain 20% more than wt synapses, again in line with high AP-2 CCV turnover. The amounts of the brain specific splice variant are however not changed. The total CCV pool of σ1B −/− synapses contains normal amounts of synaptojanin-1, the splice variant is reduced by 20% (Fig. 3A,B). This isoform lacks the AP-2 binding site and is recruited at a late stage of CCV formation via binding to e.g. endophilin and amphiphysin, suggesting isoform specific functions in the regulation of CCV uncoating^37^. However, immunoisolated CCV from σ1B −/− synapses contain 80% less of both synaptojanins than the respective wt CCV (Fig. 3A,B). Mechanisms preventing synaptojanin-1 recruitment to stabilised CCV are not isoform specific. This is a third molecular mechanism of AP-2 CCV stabilisation and thus half life extension.

Synaptojanins are recruited by binding to endophilin A1^37,38^. Endophilin A1 levels are with ~10% slightly increased in cortices and synapses of σ1B −/− mice. The total synaptic CCV pool contains 15% less endophilin A1 compared to wt CCV. However, the stabilised AP-2 CCV from σ1B −/− and wt mice contain the same amount of endophilin A1 (Fig. 3C,E). The amount of endophilin A1 does not determine the amount of synaptojanins recruited to these CCV during their formation. Their interaction appears to be inhibited.

Synaptojanin-1 is also bound by amphiphysin, which is recruited by AP-2. It stimulates dynamin and organizes the actin cytoskeleton^39–45^. Amphiphysin levels are only slightly increased in cortices and synapses of σ1B −/− mice, but it is reduced in the total synaptic CCV pool and in the immunoisolated CCV by 35% and 45% respectively (Fig. 3D,E). Thus the reduced synaptojanin-1 association is not simply a consequence of less amphiphysin recruitment. Also these data point to altered regulatory modifications.

### Co-adaptor composition of stabilised AP-2 CCV

Stabilised AP-2 CCV should represent a specific synaptic CME route, but they also may represent just an early stage in the CCV uncoating reaction. However, synaptojanin-1 data strongly indicate formation of two different classes of AP-2 CCV and thus two AP-2 endocytic routes.

To further verify that different AP-2 CCV classes and CME pathways exist, we tested for the incorporation of co-adaptor proteins AP180 and NECAP-1. AP180 is a neuron specific co-adaptor. It binds CHC with a higher affinity than AP-2 and binds cargo proteins not bound by AP-2^46^. Previously we have shown that the total synaptic AP-2 CCV pool of σ1B −/− synapses contains 30% less AP180 than wt CCV^2^. Stabilised CCV of σ1B −/− synapses contain however the same amount as the corresponding wt CCV (Fig. 3F,G). NECAP-1 regulates the interaction of AP-2 β2 with CHC by competing with CHC for the same β2 binding site, which contributes to the regulation of CCV coat polymerisation^47–49^. We had found NECAP-1 to be increased by 120% in the total synaptic AP-2 CCV pool of σ1B −/− synapses compared to wt CCV, which matches the increase in AP-2^2^. The stabilised AP-2 CCV isolated from σ1B −/− synapses contain however as much NECAP-1 as the wt CCV, despite the increase in AP-2 (Fig. 3F,G). These data confirm the existence of two CME routes within synapses.

**Figure 4:**
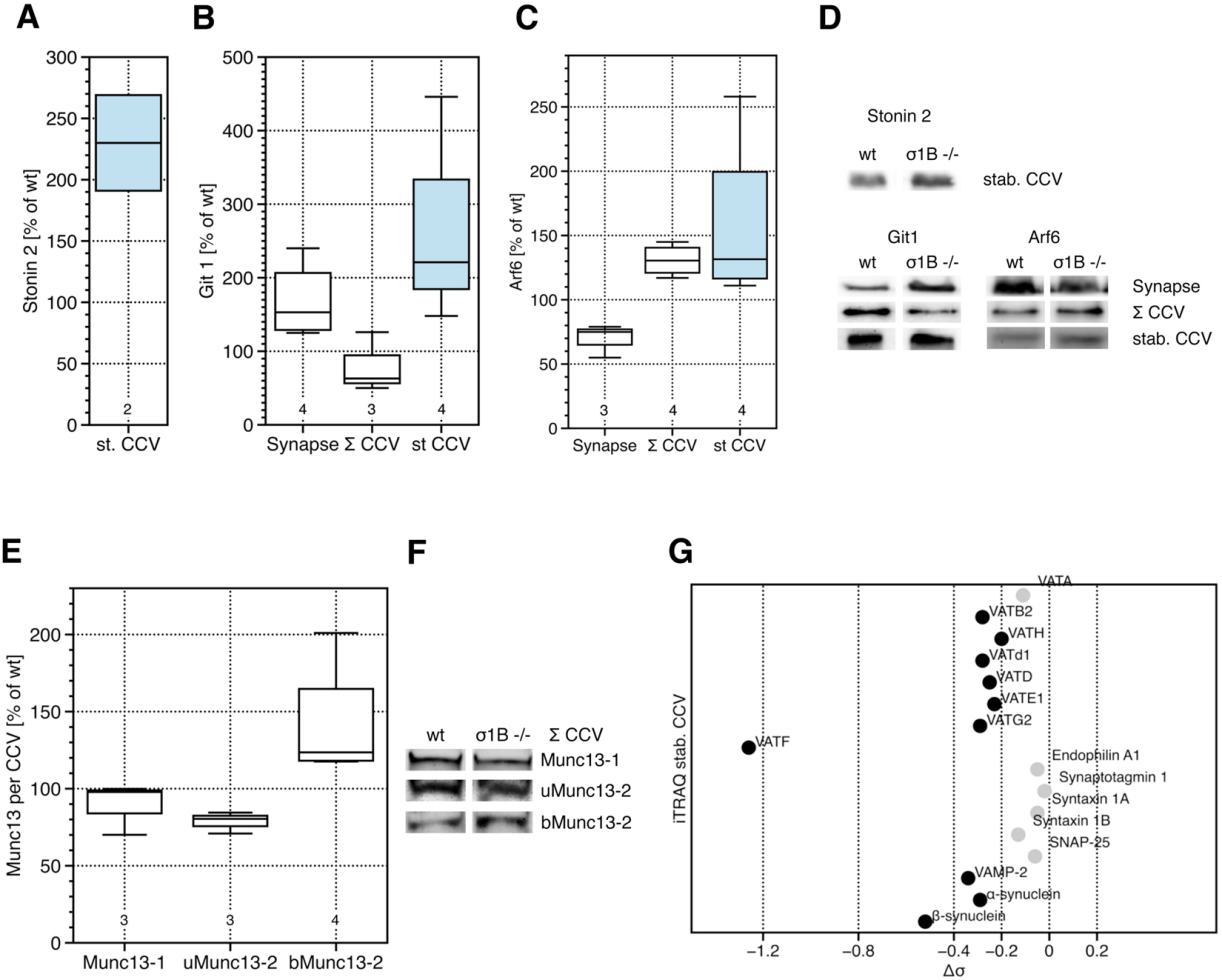
(**A**) Comparison of the association of the co-adaptor protein stonin 2 with stable CCV (st CCV, blue) isolated from σ1B −/− and wt synapses. (**B**) Comparison of the expression of the active zone protein Git1 and in (**C**) of Arf6 in σ1B −/− and wt synapses and of its association with the total pool of synaptic CCV (Σ CCV) and with the stable CCV (st CCV, blue box) from σ1B −/− and wt synapses. Quantification of semi-quantitative western-blot signals of independently prepared biological samples (wt = 100%). Numbers given below the box plots indicate the independently performed experiments. (**D**) Representative western-blot images used for the analysis shown in A and B. (**E**) Comparison of the endocytosis of the active zone Munc13 proteins by AP-2 CCV (Σ CCV) in σ1B −/− and wt synapses. Quantification of semi-quantitative western-blots of independently prepared biological samples (wt = 100%). Numbers given below the box plots indicate the independently performed experiments. (**F**) Representative western-blots of the analysis shown in A. (**G**) Comparison of the SV protein content between stable synaptic CCV isolated from σ1B −/− and wt synapses as determined by quantitative mass-spectrometry (iTRAQ). Proteins with significantly different distributions are indicated by black dots, unaltered proteins with gray dots. VAT-proteins indicate subunits of the SV V-ATPase.

Next we analysed stonin2 incorporation. It contains a μ-homology domain, homologous to the μ2 cargo binding domain. The only stonin2 cargo known to date is synaptotagmin-1, which is also sorted by SV2A/B^50–52^. σ1B −/− cortex and synapses contain ~25% and ~10% less stonin2 compared to the wt. Synaptic CCV from σ1B −/− synapses contain 20% less stonin2 than wt CCV^2^. Stabilised CCV from σ1B −/− synapses contain 130% more stonin2 than the corresponding wt CCV (Fig. 4A,D). Also these data confirm that stabilised AP-2 CCV represent a specific CME route.

### CME and AZ restructuring

If stabilised AP-2 CCV represent a specific CME route, they should be enriched in specific cargo proteins. Stonin2 is associated with Git1 (GPCR kinase-interacting protein 1), a scaffolding protein organising the cytomatrix and a presynaptic AZ structural protein. Both are arranged in circles at the outer rim of AZ. Git1 is essential for SV recycling^53^. Git1 point mutations associated with schizophrenia show defects in PAK3 (p21 protein (Cdc42/Rac)-activated kinase 3) and MAPK (mitogen-activated protein kinase) activation and signaling^54^. More stonin2 in stabilised CCV suggested that it sorts Git1 into these CCV. The total pool of σ1B −/− synaptic CCV contains 30% less Git1 than wt CCV, but stabilised AP-2 CCV contain 120% more Git1 than the corresponding wt CCV (Fig. 4B,D). Thus Git1 is redistributed from AZ into stabilised CCV, apparently mediated by stonin2. Importantly, Git1 is not endocytosed to be degraded. σ1B −/− synapses contain 50% more than wt synapses (Fig. 4B,D). Git1 is a scaffolding protein with several functional domains. It also takes part in the organisation of the cytoskeleton and the increase in synaptic levels may not be exclusively linked to its functions at the AZ^55^. Git1-deficiency leads to impaired SV recycling^53^ and thus its increased level in σ1B −/− synapses could support SV recycling, so it may partially compensate the reduction in SV numbers, suppressing the defect caused by σ1B-deficiency.

Git1 has ArfGAP activity. At the AZ its substrate is Arf6^55^. Arf6 regulates multiple vesicular trafficking routes including CME and SV recycling^56,57^ and thus we tested for AP-1/σ1B knockout induced changes in Arf6 levels. Arf6 levels are decreased by 25% in knockout synapses, but are increased by 30% in their total and in their stabilised CCV (Fig. 4C,D). This decrease is not limiting Arf6 recruitment to AP-2 CCV and Arf6 appears not to be involved in the differential regulation of the two CME pathways.

To confirm upregulation of AZ dynamics in σ1B −/− synapses we tested for enhanced CME of Munc13 proteins. We choose those because these AZ proteins bind SV and regulate SV exocytosis kinetics. Munc13 isoforms are AZ specific and are anchored to the AZ by different mechanisms^58^. We compared the content of wt and σ1B −/− synaptic CCV in Munc13-1, ubMunc13-2 and bMunc13-2. In most hippocampal synapses SV priming is solely mediated by Munc13-1, whereas ~10% contain bMunc13-2 or ubMunc13-2. σ1B −/− synaptic CCV contain wt levels of Munc13-1. ubMunc13-2 is reduced by 20% and bMunc13-2 is increased by 20% (Fig. 4E,F). Thus AZ restructuring is clearly induced by the σ1B-deficiency. Interestingly, functions of CME in AZ plasticity are AZ type specific. Future analysis of molecular mechanisms of AZ plasticity has to take this specificity into account.

**Figure 5:**
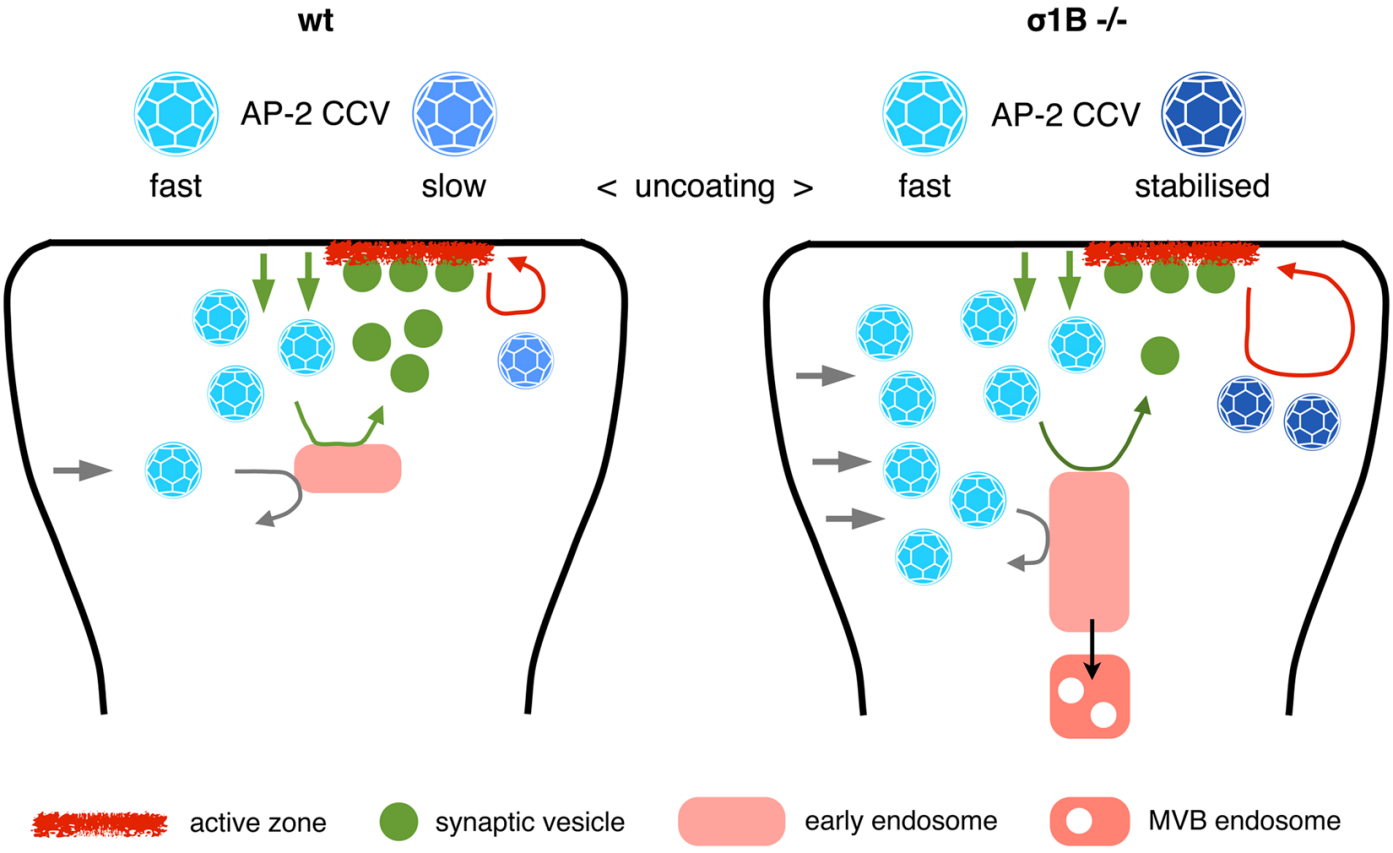
Cartoon summarises the differences between wild-type (left) and σ1B −/− (right) synapses. The functions of the different classes of AP-2 CCV in synaptic and active zone plasticity are shown. For details please refer to the discussion.

If stabilised AP-2 CCV are specialised in the regulation of AZ plasticity, SV proteins should not be enriched in these CCV. We analysed their content by quantitative protein mass spectrometry (iTRAQ) as described^2^. Stabilised CCV from σ1B −/− and wt synapses were separated in individual lanes on SDS-PAGE, lanes were cut in 24 slices, peptides were generated by trypsin and peptides from wt and σ1B −/− samples were coupled to different iTRAQ labels. In a third lane, stabilised CCV from wt and σ1B −/− synapses were mixed 1:1 and coupled to a third iTRAQ label. Peptides from those 3 lanes were mixed and coupled to a fourth iTRAQ label. Labelling with the third and fourth label should give identical numbers and only proteins which fulfilled this criterium were included in the analysis^2^. We only considered proteins with high sequence coverage and detected several SV proteins (Git1 and stonin2 peptides were not detected). The synaptotagmin-1 (sequence coverage (sc) 42.5%) content was identical in wt and σ1B −/− CCV (Fig. 5). Endophilin A1 (sc 32.4%) levels do not differ as in the semiquantitative western-blot analysis (Figs 3C,E, 4G). Syntaxin-1A and -1B, and SNAP25 (sc 20, 24.3 and 29.1%) were present at comparable amounts in CCV from both genetic backgrounds, but there was less VAMP-2/synaptobrevin-2 (sc 28.4%) in the stabilised CCV from σ1B −/− synapses compared to wt CCV. α- and β-synucleins (sc 64.3%, 31.6%) were also present and stabilised CCV from σ1B −/− synapses contained less of them. Stabilised CCV from σ1B −/− synapses also contain less V-ATPase (sc from 3.4% of VATd1, to 63.4%, of VATB2) than the corresponding wt CCV (Fig. 4G). The lower levels in SV proteins confirm the specialised function of stabilised AP-2 CCV in AZ plasticity (Fig. 5).

## Discussion

AP-1/σ1B deficiency causes severe X-linked mental retardation. Our analysis of the deficiency induced alterations in pre-synaptic protein traffic reveal novel molecular mechanisms of synaptic plasticity. In previous studies we have shown that this deficiency leads to changes in AP-1 dependent synaptic protein sorting and also induces changes in AP-2/clathrin mediated synaptic endocytosis. AP-1 mediated SV recycling is impaired leading to fewer SV, the concomitant enlargement of early endosomes and their enhanced maturation into multivesicular body endosomes. Enhanced maturation is mediated by endosomal AP-1/σ1A. This AP-1/σ1A function is inhibited by AP-1/σ1B. Alterations in SV dynamics and endosomal protein sorting are accompanied with an increase in AP-2 CCV numbers^1–3^. The increase in AP-2 CCV is a tissue and synapse specific secondary phenotype of AP-1/σ1B-deficiency. Regulation of CME is thus a mechanism of synaptic plasticity^4,6^. We describe the biochemical characterisation of the AP-1/σ1B knock-out induced synaptic CCV. Comparing their coat composition with those isolated from wt synapses revealed that their increase is due to two mechanisms: general upregulation of CME and the stabilisation of a class of these AP-2 CCV, creating AP2 CCV with an extended half life.

We demonstrate three distinct molecular mechanisms, which stabilise the CCV protein coat. In order to identify a function of stabilised AP-2 CCV we analysed their cargo protein content. They are enriched in two major AZ structural proteins, Git1 and stonin2, which assemble into rings at the rim of AZ. Stonin2 is also an AP-2 CCV co-adaptor protein, which binds Git1^53,59^ and thus may recruit Git1 into stabilised CCV. Enhanced Git1 CME by these CCV does not lead to its enhanced degradation, synaptic Git1 levels are increased demonstrating Git1 redistribution within σ1B −/− synapses. Upregulation of AZ plasticity is confirmed by the differentially altered CME of Munc13 isoforms, which regulate SV exocytosis. Thus AZ plasticity is regulated via AP-2 CCV mediated endocytosis. In contrast SV proteins are not enriched in stabilised AP-2 CCV and are preferentially sorted by the common CME pathway. This is the first demonstration of a differential regulation of AP-2 CME pathways within a cell.

We identified three distinct molecular mechanisms, which stabilise AP-2 CCV. This is the stable binding of the uncoating ATPase Hsc70, which enabled us to isolate them with anti-Hsc70 antibodies. Stable CCV from σ1B −/− synapses contain more of the Hsc70 co-chaperones auxilin1/2, but they are not able to activate Hsc70. The second mechanism is the hyperactivation of AP-2 by the enhanced recruitment of the μ2 kinase AAK1 and enhanced μ2 phosphorylation. μ2 phosphorylation stabilises AP-2 binding to cargo proteins and to PIP_2_. Both interactions lock AP-2 onto membranes. The third mechanism is the reduced recruitment of the two isoforms of the PIP_2_ phosphatase synaptojanin-1. Its activity is essential for membrane dissociation of AP-2.

Next, we have to answer the question about the molecular mechanisms controlling the differential assembly of AP-2 CCV and their half life. The clathrin-basket uncoating machinery is constitutively active, because clathrin readily self assembles in the cytoplasm without the aid of adaptor proteins building empty clathrin cages. If Hsc70 and auxilin-1/2 do not disassemble them continuously, clathrin becomes limiting for CCV formation and CME is blocked^60^. Thus during CME clathrin disassembly by Hsc70 has to be inhibited and has to be activated only after CCV have been formed. In fact, the coat of isolated CCV is unstable in physiological buffer conditions and CCV isolation requires a slightly acidic pH^23^. Cochaperone recruitment to stabilised CCV is not impaired. Interactions of auxilin-1/2 with Hsc70 are prevented, either by post-translational modifications of them or by competitive interactions of auxilins with other coat proteins. The μ2 kinase AAK1 might also regulate Hsc70 function, but our preliminary data also show alterations in additional kinases, which have not been characterised as CCV kinases previously. We have to identify their substrates to demonstrate that their functions indeed regulate CCV stability. They might also modify cargo proteins to direct them into CME. Synaptojanins can be recruited by endophilin A1, but endophilin A1 levels are not reduced in stabilised CCV. Synaptojanins bind to additional CCV coat proteins and thus the regulation of several protein-protein interactions might play a role in their reduced recruitment^37,38,61^.

The CCV immunoisolation procedure and the natural instability of CCV does not allow the precise determination of the ratio of rapid uncoating and stabilised AP-2 CCV, but their pool size can be estimated. The increases in AP-2 in total and stabilised CCV from σ1B −/− synapses are almost identical and thus their μ2-Pi ratios enable us to estimate pool size. Stabilised CCV have 215% more μ2-Pi whereas the total synaptic CCV pool has 30% more μ2-Pi than the corresponding wt pools. We can assume that the 30% increase in the total CCV pool is largely due to the increase in the stabilised pool, because the total CCV pool contains less AAK1, whereas stabilised CCV contain more AAK1. Given the 215/30 ratio, the stabilised pool represents ~15% of the total synaptic AP-2 CCV in σ1B −/− synapses. This small pool size is in line with a specialisation of these AP-2 CCV for the transport of a small subset of synaptic proteins, like the AZ proteins Git1 and stonin2.

The enrichment of AZ rim proteins in stabilised AP-2 CCV suggests that AP-2 CCV formation at the AZ rim is differentially regulated. AAK1 is not the only kinase able to phosphorylate μ2^62^. Our data indicate that a major function of AAK1 is the stabilisation of AP-2 membrane binding and thus the regulation of CCV half life. Other μ2 kinases might regulate the initial activation of AP-2 at specific plasma membrane domains. That AP-complex activation by different kinases is important for the regulation of their protein sorting functions is also indicated by the biochemistry of the AP-1 family. Differential AP-1/μ1A activation could play a role in the differential activation and thus different functions of γ2 and γ1 AP-1 complexes. In addition, the three μ1 isoforms are expected to be activated by different kinases^6,63,64^. Differential AP-2 activation could be achieved by specific targeting of kinases to the site of CME, e.g. by linker proteins or receptor kinase activities, and/or by local kinase activation through second messengers like Ca^2+^, cAMP, DAG etc. Regulation by these second messengers is an attractive model in the case of AZ. In the “shunting” model, high Ca^2+^ concentrations ([Ca^2+^]) at the AZ stimulate SV fusion and inhibit CME at the same time^65^. In the vicinity of AZ, [Ca^2+^] decreases due to diffusion and CME can take place. SV proteins have to be removed by CME to clear the AZ and to allow repeated SV fusions^8,66^. The low [Ca^2+^] affinity CME inhibitor has not been identified. Dynamin is inhibited at high [Ca^2+^], but it is only essential for membrane fission, the final step in CCV formation. However, the phosphorylation levels of CME proteins like PACSIN and intersectin increase upon stimulation of SV exocytosis with [Ca^2+^] and even more proteins are dephosphorylated^67^. Thus there is no single on/off kinase/phosphatase pair responsible for the coupling of SV exocytosis and CME. Additional regulatory mechanisms could involve the cargo proteins or their specific CCV adaptor protein. Git1 is a scaffolding protein and thus could play the role of a CCV kinome regulator^55^. Stonin2 could be involved as well. Differences in the contents of the AP-2 CCV regulator NECAP-1, but also in the membrane curvature sensing BAR-domain protein amphiphysin and the two synaptojanins, suggest that different CME routes also differ in their CCV budding kinetics, which we will study in the future.

What could be the function of CCV half life regulation and specifically its prolongation? A prolonged CCV half life will delay the fusion of these endocytic vesicles with their acceptor compartment, normally endosomes. A longer CCV half life will also allow their transport over longer distances. Coat proteins do interact with the actin cytoskeleton and AP-2 can function as a linker between cargo and motor proteins^68^. This would allow the fusion of these endocytic vesicles with organelles positioned further away from synaptic plasma membrane, e.g. late endosomes. Late endosomes are involved in slow plasma membrane protein recycling or in receptor signalling pathways. Another reason could be the upregulation of endolysosomal protein degradation in σ1B −/− synapses. Early endosomes mature into late, multivesicular body endolysosomes, which enables protein degradation. Maturation is stimulated by AP-1/σ1A and inhibited by AP-1/σ1B and in σ1B −/− synapses SV protein degradation is upregulated^2,3^. Stabilisation of AP-2 CCV allows their transport past these early endosomes and would prevent delivery of those cargo proteins in the degradative pathway. These cargo proteins can then fulfil functions somewhere else within the synapse. It would also enable their comparatively slow recycling to the plasma membrane and AZ, when required. The stabilisation of AP-2 CCV transporting Git1 indeed appears to prevent its degradation, because synaptic Git1 levels are increased in σ1B −/− synapses. However, it could also be that stabilised AP-2 CCV are not transported away from the plasma membrane and are retained in the vicinity of AZ. After their uncoating the vesicles can fuse back directly with the plasma membrane at the rim of AZ, where Git1 and stonin2 would be repositioned or they would contribute to the formation of a new AZ^53,59^. The increase in synaptic Git1 levels may be able to suppress defects in AP-1 dependent SV protein recycling, because its deficiency leads to impaired SV recycling^53^. How the various domains of Git1 effect AZ functions and SV recycling is not known and thus it is too early to develop a more detailed model about the function of Git1 containing, stabilised AP-2 CCV in AZ plasticity.

This study raises several questions, which will have to be answered in the future. How does stonin2 and Git1 sorting affect AZ function and how are these mechanisms linked to synaptic signalling? How are these AP-2 functions linked to AP-1 functions in SV protein recycling and protein degradation? Currently, we are studying the molecular mechanisms regulating the formation of long lived AP-2 CCV in synapses.

## Materials and Methods

### Isolation of synaptic CCV

The ‘knock-out’ mice have been described^1^ and also the isolation of synaptosomes and synaptic CCV has been described^2^, which followed established protocols^25^. Isolations from wt and σ1B −/− cortices were always performed in parallel. Animals used for tissue isolations were −/− animals and isogenic + / + animals derived from + /− matings. Animals are kept in the central animal facility of the Faculty of Medicine of the Georg-August-University Göttingen in accordance with the appropriate guidelines. Animals were killed with CO_2_ and cervical dislocation in accordance with the appropriate guidelines. Animal housing and the protocol for killing the animals were approved by the,Niedersächsisches Landesamt für Verbraucherschutz und Lebensmittelsicherheit‘ (LAVES). Brains were isolated from 4–6 month old animals in the afternoon, were snap frozen in liquid nitrogen and stored at −80 °C. Sucrose density gradient fractions containing the purified synaptic CCV were pooled, and incubated with protein G Sepharose bead slurry (Protein G Sepharose 4 Fast Flow GE Healthcare, Uppsala, S) at 4 °C for 1 hour. Beads were pelleted at 2000 rpm and the supernatant was incubated with 5 μg of anti-Hsc70 mouse monoclonal antibody (SYSY, Göttingen, Ger) over night at 4 °C. Protein G Sepharose beads were added at 4 °C for 4 hours. The harvested beads were washed twice with CCV buffer and resuspended in 40μL 3x SDS-PAGE loading buffer. The protein content of the beads and the wash fractions elution 1 and elution 2 were analysed by semi-quantitative western-blot analyses and protein mass spectrometry.

### CCV coat protein quantification

Brain extracts were prepared from wt and ‘ko’ mice in parallel and comparisons of protein content were only made between extracts prepared in parallel. Comparing the data from different animals and independent preparations requires a normalisation and thus wt values were defined as 100%. The protein load was varied between 10–80 μg per lane to determine the linear protein/chemiluminescence signal ratio. Several proteins of different molecular masses were detected on one western-blot nitrocellulose membrane. This served as internal control for protein isolation and detection by the ECL luminescence detection kit PICO (Pierce-ThermoScientific, Karlsruhe, Ger), recorded with a Fuji LAS 1000 (Fujifilm Corp., Düsseldorf, Ger) camera system. Statistics of the quantifications are presented as Bar-plot diagrams, which were generated using DataGraph (Visual Data Tools, USA). Protein determination: Bradford-assay (BioRad, Munich, Ger). Antibodies: anti-α and anti-γ1 adaptin (1:2000) were from BD Biosciences; anti-CHC (1:2000) was from translab; anti-CLC (1:5000), anti-dynamin (1:1000), anti-CSPα (1:1000), anti-Hsc70 (1:500), anti-AP180 (1:5000), anti-Amphiphysin (1:1000), anti-synaptojanin 1 (1:500), its splice-variant (1:500) and anti-endophilin A1 (1:1000) were from Synaptic Systems; anti-auxilin 1 (1:600), anti-Arf6 (1:1000) and anti-NECAP1 (1:200) were from proteintech; anti-auxilin 2 (1:200) was from Santa Cruz; anti-Git1 (1:1000) was from Acris; anti-AAK1 (1:100) was a generous gift by S. Connor & S. Schmidt (University of Minnesota, USA & University of Texas Southwestern Medical Center, Dallas, USA); anti-μ2-Pi (1:500) was a generous gift from S. Höning (University Cologne, Ger); anti-stonin 2 (1:500) was a generous gift by V. Haucke (FMP Berlin, Ger); anti-Munc13 antisera (1:500) were a generous gift by N. Brose (MPI for Experimental Medicine, Göttingen, Ger). HRP-conjugated antibodies (1:10000): Dianova (Hamburg, Ger), anti-mouse (product # 111-035-144), anti-rabbit (product # 115-035-062) anti-goat (product # 305-035-045).

### Quantitative protein mass spectrometry

Proteins separated by SDS-PAGE were subjected to an automated workflow comprising in-gel digestion and subsequent labelling of the tryptic peptides with iTRAQ reagents as already published and described in great detail^2,69^. Labeled peptides were separated by nano-flow liquid chromatography, spotted onto AnchorChip MALDI targets, and analysed on an Ultraflextreme MALDI-TOF/TOF mass spectrometer (Bruker, Bremen, Ger). Signals were normalised to the control (wt + ‘ko’) and signal ratios of each sample were normalised to the average deviation of the sample to correct for variations. Values within the standard deviation (variance) ± σ were considered insignificant.

## Electronic supplementary material

Supplementary Information
