# Supplementary material for: Differential regulation of synaptic AP-2/clathrin vesicle uncoating in synaptic plasticity
Source: Sci Rep. 2017 Nov 17;7:15781. doi: 10.1038/s41598-017-16055-4 (PMC5694008; doi:10.1038/s41598-017-16055-4)
Supplement: Supplementary file 1 — Supplementary Information [file 41598_2017_16055_MOESM1_ESM.pdf]

Supplementary information for the article:

Differential regulation of synaptic AP-2/clathrin vesicle uncoating in synaptic plasticity

by

Ermes Candiello, Ratnakar Mishra, Bernhard Schmidt, Olaf Jahn and Peter Schu

Images show the western-blot membranes which were used to show representative band signal intensities in the figures of the main manuscript.
Please refer to the results and materials & methods section of the main manuscript for informations concerning the experimental design and experimental details.

AP-2 α-adaptin

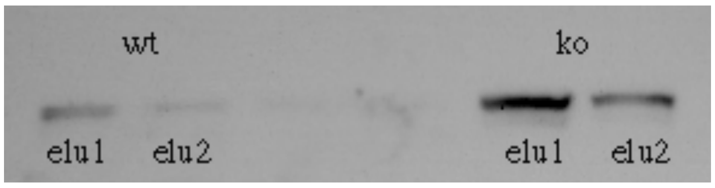

AP-1 γ1-adaptin

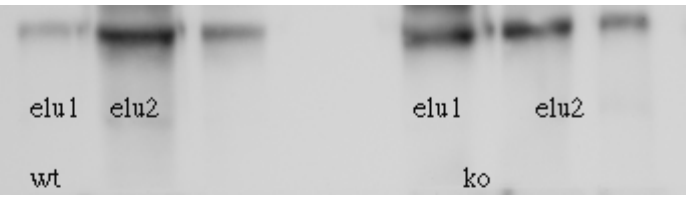

Western-blot signals of immunoisolated synaptic CCV elution fractions

Clathrin-heavy-chain CHC

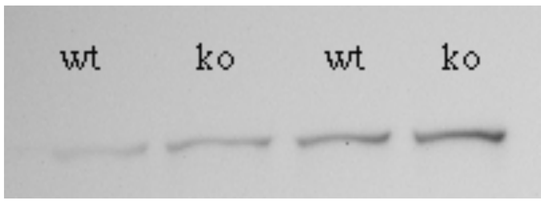

Clathrin-light-chain CHC

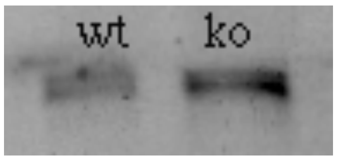

CSPα

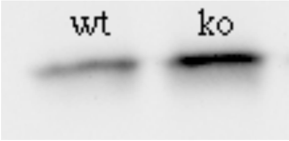

Dynamin

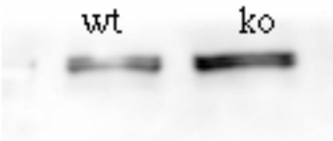

Western-blot signals of immunoisolated synaptic CCV pooled proteins

Dynamin in the total synaptic CCV pool

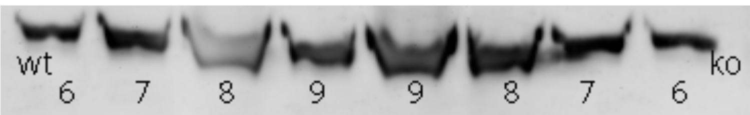

Western-blot signals of the density gradient fractions (fraction numbers) containing synaptic CCV

Hsc70

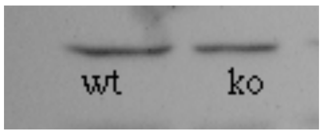

auxilin 1

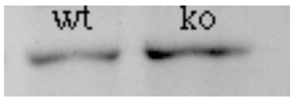

Western-blot signals of immunoisolated synaptic CCV pooled proteins

auxilin 2

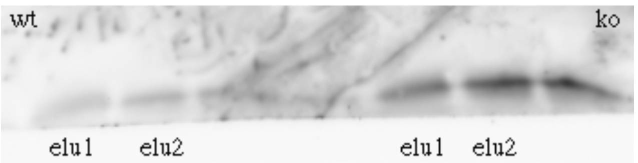

Western-blot signals of immunoisolated synaptic CCV elution fractions

phosphorylated µ2 adaptin of AP-2

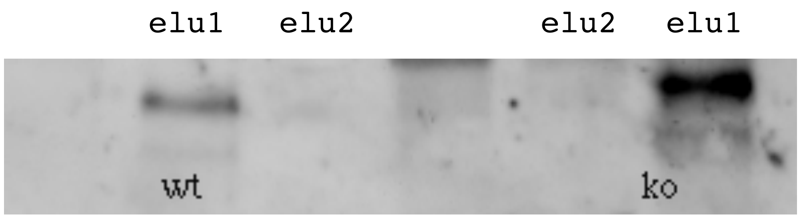

AAK1

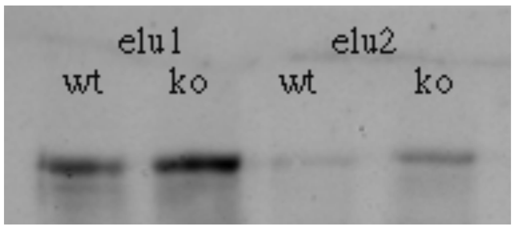

Western-blot signals of immunoisolated synaptic CCV elution fractions

Synaptojanin 1

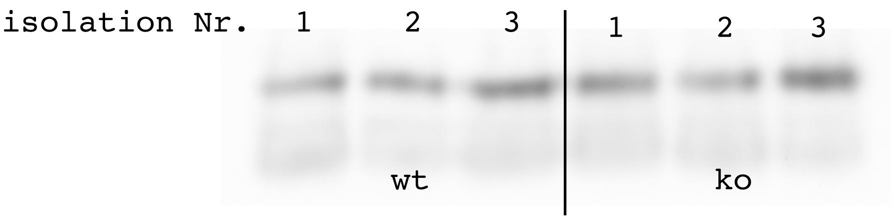

Synaptojanin 1 splice variant

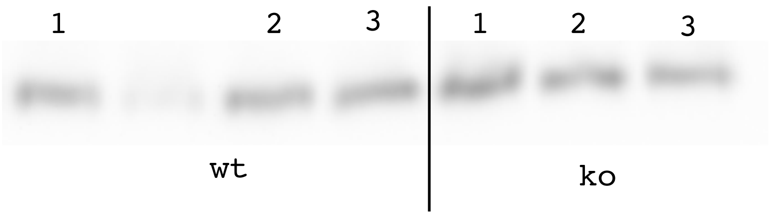

Western-blot signals of cortices isolated from 3 wt and 3 ko mice in parallel.

Synaptojanin 1

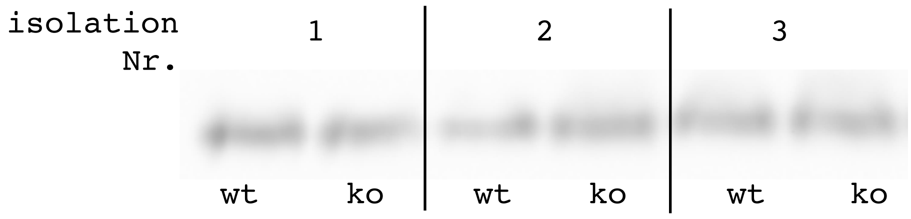

Synaptojanin 1 splice variant

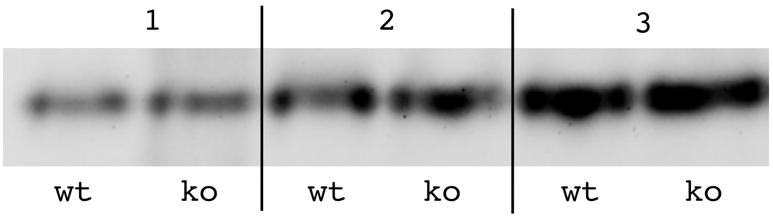

Western-blot signals of synapses isolated from 3 wt and 3 ko mice in parallel.

Synaptojanin 1

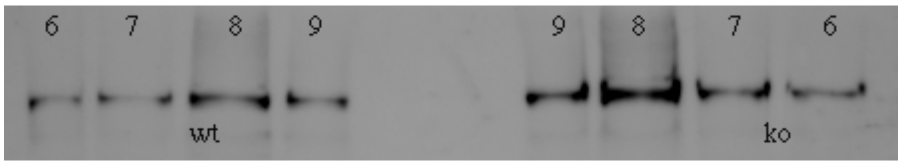

Synaptojanin 1 splice variant

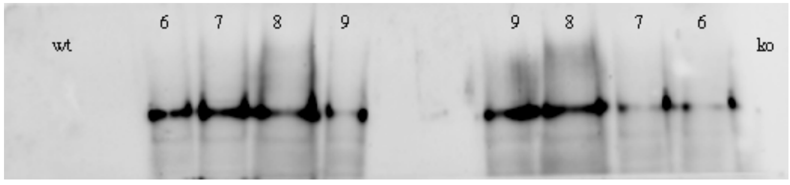

Western-blot signals of the density gradient fractions (fraction numbers) containing synaptic CCV

Synaptojanin 1

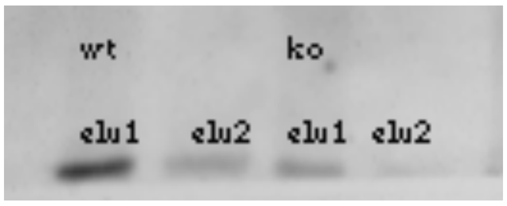

Synaptojanin 1
splice variant

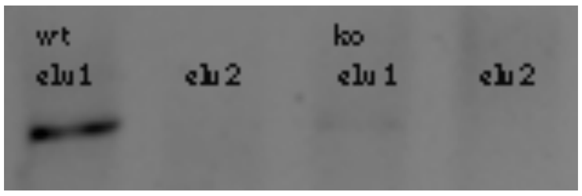

Western-blot signals of immunoisolated synaptic CCV elution fractions

Endophilin A1

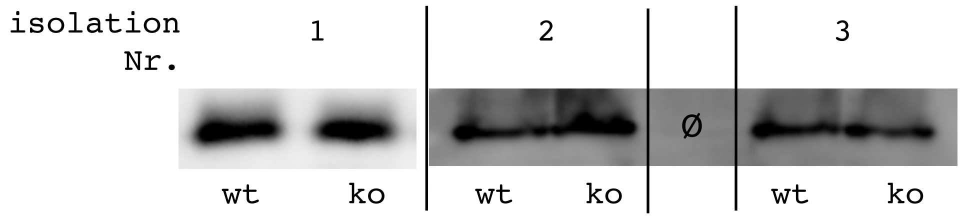

Amphiphysin

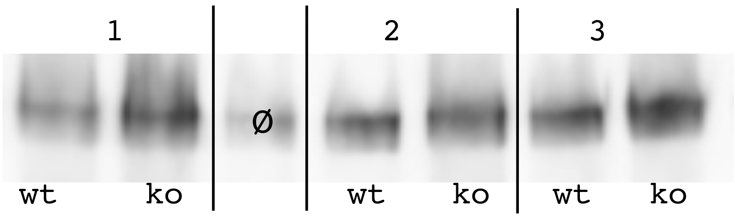

Western-blot signals of cortices isolated from 3 wt and 3 ko mice in parallel.

Endophilin A1

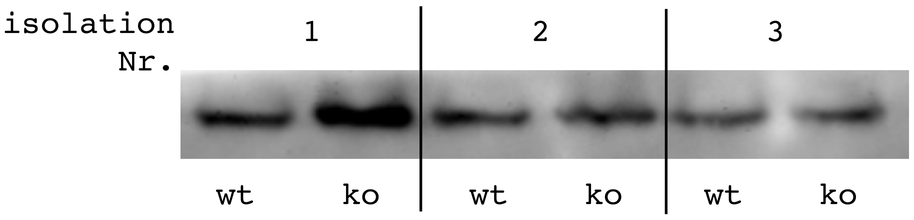

Amphiphysin

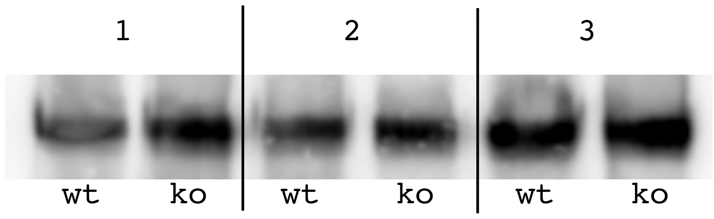

Western-blot signals of synapses isolated from 3 wt and 3 ko mice in parallel.

Endophilin A1

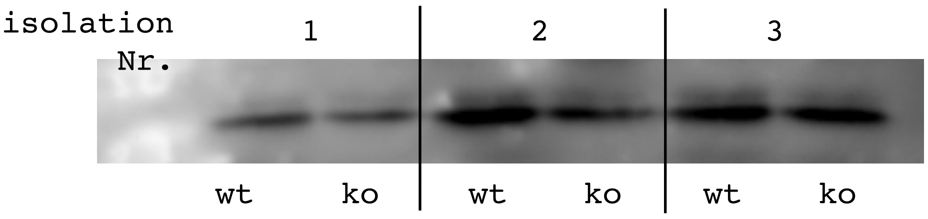

Western-blot signals of synaptic CCV (pooled gradient fractions) isolated from 3 wt and 3 ko mice in parallel.

Amphiphysin

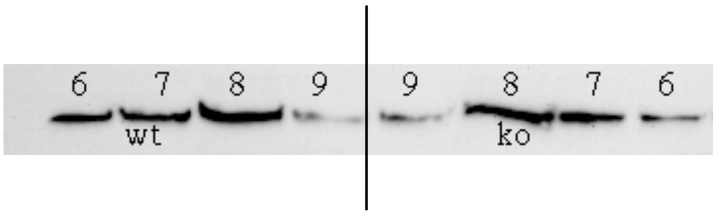

Western-blot signals of the density gradient fractions (numbers indicate gradient fraction numbers) containing synaptic CCV.

Endophilin A1

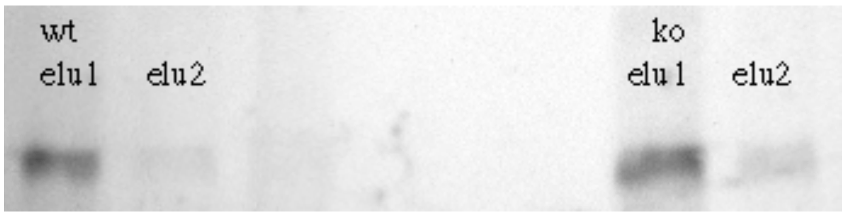

Western-blot signals of immunoisolated synaptic CCV elution fractions

Amphiphysin

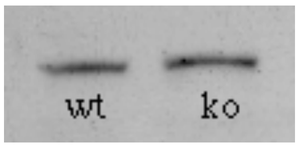

Western-blot signals of immunoisolated synaptic CCV pooled elution fractions

AP180

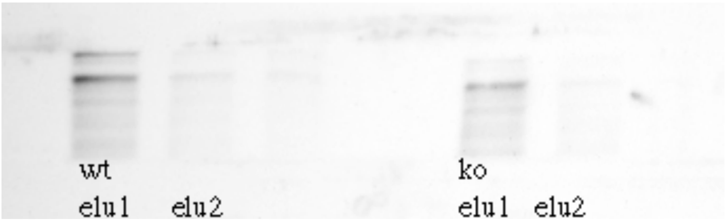

Western-blot signals of immunoisolated synaptic CCV elution fractions

Stonin 2

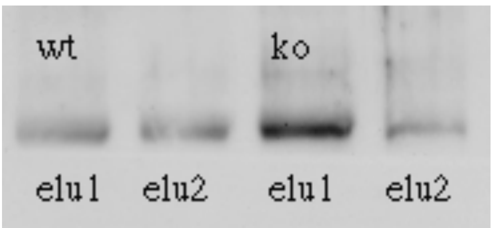

Western-blot signals of immunoisolated synaptic CCV elution fractions

Git1

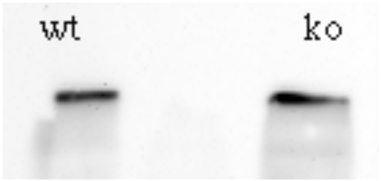

Western-blot signals of immunoisolated synaptic CCV pooled elution fractions

NECAP1

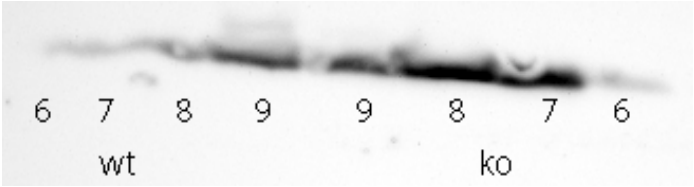

Western-blot signals of the density gradient fractions (numbers indicate gradient fraction numbers) containing synaptic CCV.

NECAP1

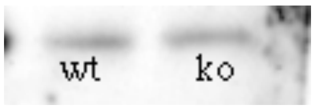

Western-blot signals of immunoisolated synaptic CCV pooled elution fractions

Arf6

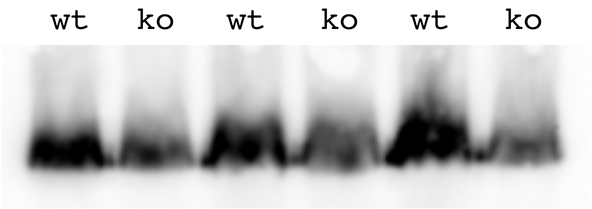

Western-blot signals of synapses isolated from 3 wt and 3 ko mice in parallel.

Arf6

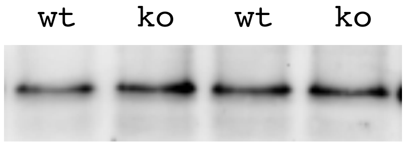

Western-blot signals of immunoisolated synaptic CCV pooled gradient fractions

Arf6

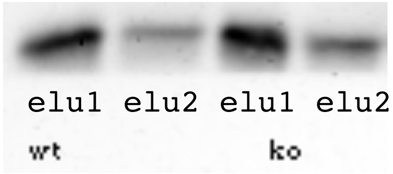

Western-blot signals of immunoisolated synaptic CCV elution fractions

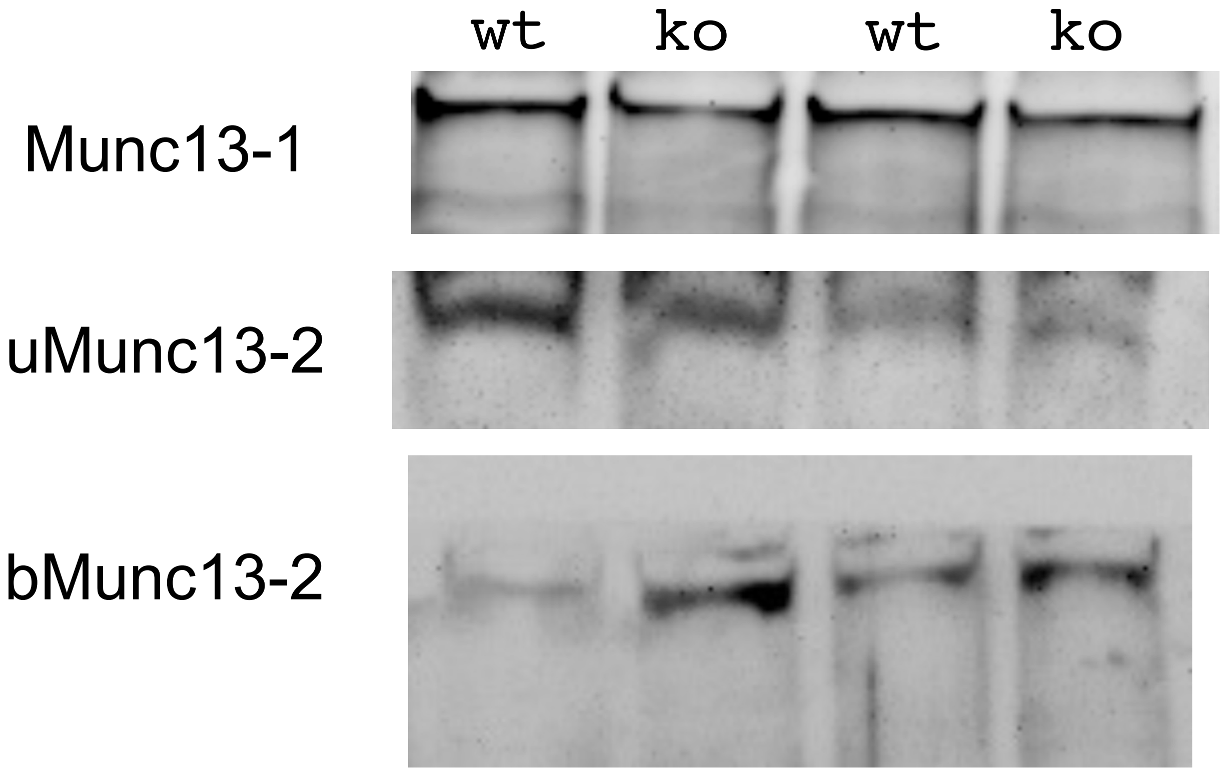

Western-blot signals of synaptic CCV pooled gradient fractions prepared from 2 wt and 2 ko mice in parallel.
